# Heterogeneous clinicopathological findings and patient-reported outcomes in adults with *MN1*-altered CNS tumors: A case report and systematic literature review

**DOI:** 10.3389/fonc.2023.1099618

**Published:** 2023-01-19

**Authors:** Stephen C. Frederico, Elizabeth Vera, Zied Abdullaev, Alvina Acquaye, Kenneth Aldape, Lisa Boris, Nicole Briceno, Anna Choi, Alexa Christ, Diane Cooper, Ewa Grajkowska, Tricia Kunst, Heather E. Leeper, Jason Levine, Nicole Lollo, Drew Pratt, Martha Quezado, Ritu Shah, Kathleen Wall, Mark R. Gilbert, Terri S. Armstrong, Marta Penas-Prado

**Affiliations:** ^1^ Neuro-Oncology Branch, Center for Cancer Research (CCR), National Cancer Institute (NCI), National Institutes of Health (NIH), Bethesda, MD, United States; ^2^ University of Pittsburgh School of Medicine, Pittsburgh, PA, United States; ^3^ Laboratory of Pathology, National Cancer Institute (NCI), National Institutes of Health (NIH), Bethesda, MD, United States; ^4^ Office of Research Services, National Institutes of Health (NIH), Bethesda, MD, United States; ^5^ Pediatric Oncology Branch, Center for Cancer Research (CCR), National Cancer Institute (NCI), National Institutes of Health (NIH), Bethesda, MD, United States; ^6^ IT and Clinical Informatics, Center for Cancer Research (CCR), National Cancer Institute (NCI), National Institutes of Health (NIH), Bethesda, MD, United States; ^7^ Department of Radiology and Imaging Sciences, National Cancer Institute (NCI), National Institutes of Health (NIH), Bethesda, MD, United States

**Keywords:** CNS, HGNET-*MN1*, astroblastoma, MN1, rare CNS tumor, adult

## Abstract

The uncommon *MN1*-altered primary central nervous system (CNS) tumors were recently added to the World Health Organization 2021 classification under the name Astroblastoma, *MN1*-altered. Another term used to describe them, “High-grade neuroepithelial tumor with *MN1* alteration” (HGNET-MN1), makes reference to their distinct epigenetic profile but is currently not a recommended name. Thought to occur most commonly in children and predominantly in females, *MN1*-altered CNS tumors are associated with typical but not pathognomonic histological patterns and are characterized by a distinct DNA methylation profile and recurrent fusions implicating the *MN1* (meningioma 1) gene. Diagnosis based on histological features alone is challenging: most cases with morphological features of astroblastoma (but not all) show these molecular features, whereas not all tumors with *MN1* fusions show astroblastoma morphology. There is large variability in reported outcomes and detailed clinical and therapeutic information is frequently missing. Some patients experience multiple recurrences despite multimodality treatment, whereas others experience no recurrence after surgical resection alone, suggesting large clinical and biological heterogeneity despite unifying epigenetic features and recurrent fusions. In this report, we present the demographics, tumor characteristics, treatment, and outcome (including patient-reported outcomes) of three adults with *MN1*-altered primary CNS tumors diagnosed *via* genome-wide DNA methylation and RNA sequencing. All three patients were females and two of them were diagnosed as young adults. By reporting our neuropathological and clinical findings and comparing them with previously published cases we provide insight into the clinical heterogeneity of this tumor. Additionally, we propose a model for prospective, comprehensive, and systematic collection of clinical data in addition to neuropathological data, including standardized patient-reported outcomes.

## Introduction

The term high-grade neuroepithelial tumor with *MN1* alteration (HGNET-MN1) was first introduced in 2016 as one of four novel tumors with a distinct DNA methylation profile that emerged after analysis of CNS PNETs (central nervous system primitive neuroectodermal tumors) (1, 2). In addition, the presence of a recurrent fusion implicating the *MN1* (meningioma 1) gene was described; however, the specific mechanism by which *MN1* fusions drive tumor development is still unknown (1, 3). Due to histopathological overlap with other CNS tumors and a lack of a uniform immunohistochemical profile, the diagnosis of MN1-altered tumors with conventional methods remains challenging. Quite often, tumors with the HGNET-MN1 epigenetic profile are diagnosed histologically as either ependymomas or astroblastomas (1, 2), and most tumors histologically diagnosed as astroblastoma belong to this molecular entity (2). In recognition of this association, the 2021 World Health Organization (WHO) classification of CNS Tumors updated the previous morphological diagnosis of astroblastoma to reflect these molecular findings, and renamed this tumor as astroblastoma, *MN1*-altered, simultaneously recommending against the use of the term HGNET-MN1. However, this latest WHO classification also comments on the need for future work to establish clear histopathological and molecular criteria by which astroblastomas with *MN1* alterations can be distinguished from morphologically comparable neuroepithelial tumors with similar genetic alterations (4). In most cases this tumor arises in the supratentorial region and is thought to primarily affect children (1, 3). Unfortunately, available clinical and outcome data is quite limited due to the rarity of this tumor. Additionally, there is a lack of universally available molecular testing required for diagnosis which creates a challenge for retrospective data collection. Few publications provide information about the clinical outcomes for patients diagnosed with astroblastoma MN1-altered or HGNET-MN1, collectively referred to within this manuscript as MN1-altered CNS tumors.

Given their rarity, recent description, and heterogeneous outcome, evidence-based treatment guidelines are not available and prospective studies that would help determine optimal therapy are difficult to implement. In this report, we present three patients with *MN1*-altered CNS tumors, two initially diagnosed as young adults, and one presenting with a late recurrence after being diagnosed in childhood. *MN1*-altered CNS tumors in adults are even a more uncommon clinical presentation for which only anecdotal experience has been reported to date.^6–7,12,15^ In addition to discussing these adult patients’ demographics, clinical presentation, imaging, pathological and molecular findings, we also discuss their treatment and outcome, including patient-reported outcomes (PROs). Our cases and those previously reported highlight the clinical heterogeneity of this tumor and potential management options. Additionally, we propose a model for longitudinal, comprehensive, and systematic collection of clinical data, including standardized patient-reported outcomes, that is aimed at improving the understanding of these and other newly described rare CNS tumors for which prospective clinical data is often impossible to obtain, and retrospective data collection is often limited to basic demographic and survival data.

## Methods

The three cases discussed in this manuscript were retrieved from enrollees in an IRB-approved Natural History Study (NHS) at the Neuro-Oncology Branch, National Cancer Institute (NCI), National Institutes of Health (NIH) (NCT02851706), which allows longitudinal evaluation of adult patients with primary CNS tumors who are probable future candidates for NCI Phase I and II protocols, including tumors that are understudied or have indeterminate natural history. Of 950 participants enrolled in this study as of October 21, 2021, only three were found to be diagnosed with *MN1*-altered tumors. NHS longitudinal data collection includes comprehensive, structured clinical information, PROs (MDASI-BT and/or SP, Patient-Reported Outcome Measurement Information System [PROMIS], NeuroQOL Cognition Function, and EQ-5D-3L) completed at baseline and each subsequent clinic visit, and collection of peripheral blood and tumor tissue samples.

### Patient-reported outcomes

Symptom occurrence has been shown to predict treatment course and survival in patients with solid tumors. PROs provide information directly from patients about their symptoms, quality of life, and functional status associated with their tumor and/or treatment. PROs can help optimize patient care, provide information that goes beyond mere survival, and have not been previously reported for those diagnosed with *MN1*-altered CNS tumors.

The MD Anderson Symptom Inventory-Brain Tumor (MDASI-BT) is a validated instrument that allows self-reporting of symptom severity and interference with daily activities (5). The inventory utilizes a scoring system with 0 representing a symptom which was not present/did not interfere with the patient’s life over the last 24 hours, to 10 which represents a symptom that is as bad as you could imagine and interfered completely with a patient’s life. Scores of greater than or equal to 5 are considered moderate to severe.

Emotional state was assessed with “PROMIS-depression short form 8a” and “Anxiety short form 8a.” Mean *t*-scores for depression and anxiety were calculated based on the general U.S. population mean *t*-score of 50. Mean *t*-scores greater than 60 indicate moderate to severe depression or anxiety.

Neuro-QoL Cognition Function measures self-perceived cognition; a mean *t*-score of 50 is considered average for the general U.S. population. Mean *t*-scores less than 40 are considered moderate to severe impairment of cognitive function.

EQ-5D-3L measures general health status. Responses can be described as health states, where each digit of the five-digit health state reflects the severity of its corresponding dimension. The “11111” health state reflects no problems in any dimension. An index score is calculated based on U.S. population weights and reflects the patient’s perception of their own health. An index score of 1 indicates the patient perceives their health as perfect; an index score of 0 indicates they perceive their health as bad as death; and a negative index score indicates they perceive their health as worse than death.

### Tumor testing

All tumor specimens were reviewed histologically, and molecular analysis was performed at the Laboratory of Pathology, NCI, Bethesda, Maryland. Methods are summarized in **Supplementary Tables 4, 5**. Final interpretation of each case was based on integration of methylation-based classification, histopathological findings, clinical history, and data reported in the biomedical literature.

### Literature review

We performed a systematic review of the literature following the Preferred Reporting Items for Systematic Review and Meta-Analysis (PRISMA). In consultation with an NIH biomedical research librarian, a search was performed in PubMed (NLM), EMBASE (Elsevier), Web of Science (Clarivate), and Scopus (Elsevier) from December 2022 back to the start of each database. Due to few studies, we searched broadly for studies using only the key terms HGNET-MN1, astroblastoma, and MN1-altered in the topic fields and in the title/abstract fields. PubMed retrieval was 16 citations; EMBASE retrieval was 36 citations, Web of Science retrieval was 18 citations, and Scopus retrieval was 13 citations (**Supplementary Figure 2**). The total from all four databases was 83 citations. Of the 83 citations, 43 citations were duplicates. The remaining 40 citations were reviewed in full text based on our inclusion and exclusion criteria. Studies were included if they discussed the clinical management of the initial malignancy, provided patient OS and PFS, and there was a molecular diagnosis of HGNET-MN1 or astroblastoma-MN1 altered, at a minimum. Studies were excluded either due to there being a lack of molecular and/or detailed clinical data (reason 1), or due to the tumor being an extracranial lesion (reason 2). Of the 40 citations reviewed in full text, 9 of these studies were included in our manuscript, collectively describing 27 cases.

#### Patient 1

The patient is a 25-year-old female who was first diagnosed with a left fronto-parietal tumor at 5 years of age after presenting emergently with right-sided facial weakness and urinary incontinence. She underwent surgical resection outside the United States, and pathology was informed as “ependymoma”. The tumor recurred when she was 7 years old, and she underwent additional surgical resection at that time and again one year later, then receiving 59.4 Gy involved brain field radiotherapy. After completing radiotherapy, she experienced her first seizure.

**Figure 1:**
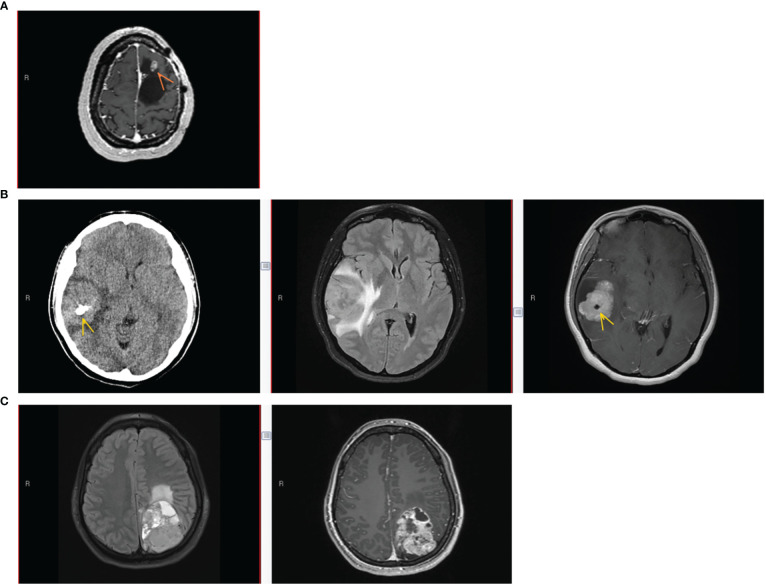
Patient imaging findings. **(A)** Patient #1 imaging findings at 3^rd^ recurrence. Axial T1-weighted MRI with gadolinium showing an enhancing nodule (arrow) along the anterior-superior margin of the previous left frontal resection cavity. **(B)** Patient #2 imaging at initial presentation. Left to right: Axial CT without contrast, Axial T2/FLAIR MRI, and Axial T1-weighted MRI with gadolinium. Lobular homogeneously enhancing mass with cystic component in the right frontotemporal region, with vasogenic edema, mass effect, midline shift, and intratumoral calcification (arrow). **(C)** Patient #3 imaging at initial presentation. Left to right, Axial T2/FLAIR MRI, and Axial T1-weighted MRI with gadolinium. Large heterogeneously enhancing left parieto-occipital mass with perilesional edema and midline shift.

**Figure 2:**
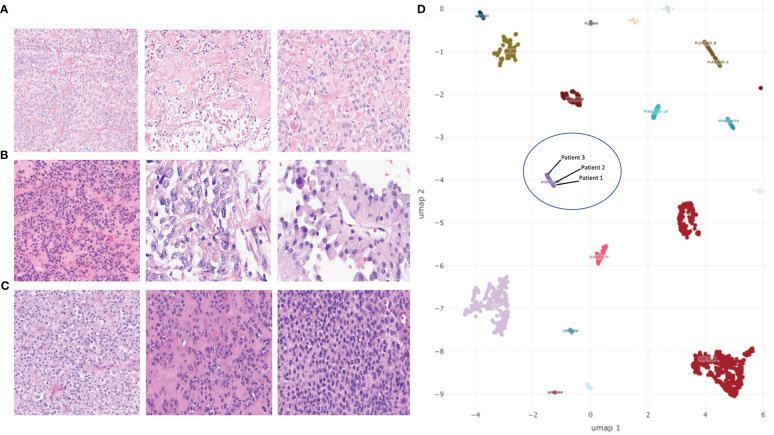
H&E-stained sections of tumors and UMAP embedding of DNA methylation array data for select tumor types. **(A–C)** H&E-stained sections showing the range of histologic characteristics of astroblastoma (wide-tapered perivascular pseudorosettes and prominent hyalinization) for patient 1 **(A)**, patient 2 **(B)**, and patient 3 **(C)**. **(D)** UMAP embedding of *MN1-*altered astroblastoma samples (HGNET-MN1), embedded among select groups of CNS tumors. Three samples from *MN1*-fused patients discussed in this study are included in a blue circle.

Her imaging remained stable for 16 years until medically refractory epilepsy prompted repeat imaging. A brain MRI with and without gadolinium at age 24 revealed a right fronto-parietal enhancing nodule within the resection cavity (**Figure 1A**). She underwent a gross total resection of this new enhancing nodule. Histopathology showed a tumor with moderate cellularity, composed of monomorphic cells with round/oval nuclei and rhabdoid appearance. Mitoses were rare. No areas of hemorrhage or tumor necrosis were observed. The tumor had discrete interface with surrounding brain parenchyma (**Figure 2A**). The neoplastic cells were positive for GFAP (subset) and INI (retained); while negative for L1CAM, NeuN, and BRAF V600E. Olig2 staining was not performed. Synaptophysin showed focal, often dot-like staining, of unclear significance; Ki67 proliferation index was low. Additional molecular analysis including genome-wide DNA methylation and gene panel *via* Oncomine Comprehensive Assay v3 were performed. Methylation class was “CNS high-grade neuroepithelial tumor with *MN1* alteration” (**Figure 2D**). MGMT promoter methylation was negative. Copy number variations can be found in **Supplementary Figure 1**. *SLX4* (p.Ala1755Val) and *ESR1* (p.Leu23Val) mutations were identified and classified as variants of uncertain significance (variant allele frequency 52.9% and 49.3%, respectively). Homozygous CDKN2A deletion was not observed. RNA sequencing was performed as a research test and a *MN1::BEND2* fusion was found. Tissue from the patient’s two prior recurrences during her childhood were analyzed *via* DNA methylation and found to be also consistent with HGNET-MN1.

Following her most recent resection, she continued MRI surveillance, and her imaging has remained without evidence of recurrent or residual tumor for the last 3 years. Her most recent neurologic exam revealed weakness of her right hand and foot. She described having partial seizures occurring typically twice per month and lasting from 1 to 10 minutes, and generalized seizures occurring typically twice per year. She was receiving levetiracetam and lacosamide. In contrast with the stable imaging findings, PROs obtained during surveillance revealed seven symptoms rated as moderate to severe and with moderate to severe interference with daily activities (MDASI-BT), moderate to severe depressive symptoms (PROMIS), and an index score of 0.67 on EQ-5D-3L with some problems with walking and self-care and moderate pain/discomfort and moderate anxiety/depression (**Table 1**; **Supplementary Tables 1–3**).

**Table 1:** MDASI-BT measurement of symptom burden and interference for Patients 1–3.

	Patient 1	Patient 2	Patient 3
Timing	Surveillance	Surveillance	Treatment initiation
Overall symptom factor	3.7	1.4	3.4
Affective factor	8.4	1.6	5.2
Cognitive factor	1.5	2.8	4.5
Neurologic factor	4.5	0.8	3.5
Treatment-related factor	4.0	1.3	2.0
General disease factor	1.0	1.0	2.5
Gastrointestinal factor	0.0	0.0	0.5
Number of moderate-severe symptoms	7	0	6
Overall interference	5.3	1.0	5.8
Activity-related interference	4.0	1.3	6.7
Mood-related interference	6.6	0.7	5.0
Moderate-severe interference	Yes	No	Yes

#### Patient 2

This patient is a 29-year-old right-handed female who presented 2 years prior with worsening headaches, nausea and vomiting. A brain MRI revealed a right frontal-temporal mass (**Figure 1B**). She underwent surgical resection with pathology revealing a tumor composed of epithelioid cells forming vague rosettes and with patchy hyalinization of vessel walls, suggestive of a morphological diagnosis of astroblastoma (**Figure 2B**). No areas of hemorrhage or tumor necrosis were observed. The tumor was diffusely positive for vimentin but showed only patchy stain for GFAP. Only rare cells were positive for S-100. There was cytoplasmic staining for EMA, and synaptophysin showed dot-like positivity in the cytoplasm of tumor cells. Neuron-specific enolase also showed patchy staining within the tumor. INI-1 staining was retained by the tumor cells, and Olig2 staining was negative. Immunohistochemical stain for Ki-67 demonstrated a focal elevated labeling index of 10%–20%. Additional molecular analysis included genome-wide DNA methylation and next generation sequencing using a Primary CNS Tumor panel. Methylation class was “CNS high-grade neuroepithelial tumor with MN1 alteration” (**Figure 2D**). Copy number variations can be found in **Supplementary Figure 1**. *PARP1* (p.Trp481Cys) and *RB1* (p.Gin436Lys) mutations of uncertain significance with variant allele frequency of 53% and 49%, respectively, were identified. Homozygous CDKN2A deletion was not observed. RNA sequencing *via* CLIA testing revealed a *MN1::BEND2* fusion.

Following tumor resection, she underwent involved-field radiotherapy (unknown dose) with concurrent temozolomide completed 3 months after resection. Her imaging remained stable without evidence of recurrent or residual tumor at her last visit 2 years after treatment. As of the patient’s most recent physical exam, her KPS score was rated as 90 with no major neurological deficit. PROs obtained during surveillance revealed no symptoms rated as moderate to severe, no moderate to severe interference with daily activities (MDASI-BT), no moderate to severe depressive or anxiety symptoms (PROMIS), and an index score of 1.00 on EQ-5D-3L (**Table 1**; **Supplementary Tables 1–3**). She was not on any medications and continues brain MRI surveillance every 4–6 months.

#### Patient 3

This patient is a 36-year-old female who had presented emergently 3 years prior reporting bilateral headaches, right-sided blurry vision, and intermittent diplopia. She had right incomplete homonymous hemianopsia and a right cranial nerve VI palsy on exam. A brain MRI revealed a left parieto-occipital enhancing mass (**Figure 1C**). She underwent a complete surgical resection and outside pathology was reported as “CNS embryonal tumor with rhabdoid features”. Histopathology revealed a malignant and infiltrative neoplasm composed of sheets of cells with abundant eosinophilic to clear cytoplasm, eccentric nuclei, and prominent nucleoli with associated areas of hemorrhage and tumor necrosis, but also perivascular pseudorosettes (**Figure 2C**). The neoplastic cells were positive for vimentin and glypican3 while negative for synaptophysin, GFAP, keratin and inhibin. EMA staining was focally positive. Olig2 staining was not performed. DNA methylation performed in two independent CLIA certified laboratories revealed a methylation class of “CNS high-grade neuroepithelial tumor with MN1 alteration” (**Figure 2D**). Copy number variations can be found in **Supplementary Figure 1**. No clinically relevant variants were detected *via* Primary CNS Tumor gene panel. Homozygous CDKN2A deletion was not observed. RNA sequencing *via* CLIA testing revealed a *MN1::BEND2* fusion.

She received craniospinal radiation to a total dose of 30.6 Gy in 17 fractions with a 30 Gy boost in 15 fractions to the left parietal surgical bed completed 3.5 months following resection. She experienced her first disease progression 7 months after the initial tumor resection and began receiving cisplatin and lomustine. She required dose reduction after 3 cycles due to myelosuppression. About 12 months following her initial diagnosis imaging showed a second tumor progression. She then underwent a second gross total resection followed by Gamma Knife radiosurgery (5 fractions to the left parietal region; dose unknown). Seven months later, imaging showed enlarging enhancement adjacent to the left parietal cavity with increased perilesional edema and enhancement extension into the craniotomy defect. Another subtotal resection confirmed tumor recurrence. She began exploring potential clinical trials at our center.

At the time of her first visit, her KPS score was rated as 80. She had dysarthric speech, a slow and cautious gait and was unable to perform tandem gait. She was on multiple medications including dexamethasone, oxycodone, docusate, pantoprazole, levetiracetam, ondansetron, aripiprazole, trazadone, benzonatate, albuterol inhaler, melatonin, and acetaminophen.

Her MRI revealed nodular contrast enhancement in the walls of the most recent resection cavity. She was tapered off dexamethasone and enrolled in a clinical trial on which she began receiving a checkpoint inhibitor. PROs obtained prior to treatment initiation revealed 6 symptoms rated as moderate to severe and with moderate to severe interference with daily activities (MDASI-BT), moderate to severe depressive symptoms (PROMIS), moderate to severe impairment in perceived cognition, and an index score of 0.71 on EQ-5D-3L with some problems with walking and performing usual activities, moderate pain/discomfort, and moderate anxiety/depression (**Table 1**; **Supplementary Tables 1–3**). Two months after starting experimental treatment her MRI showed increased T1 contrast enhancement in the occipital resection site with associated T2/FLAIR hyperintensity, extending into the left thalamus and left parietal lobe. With this imaging progression, the patient stopped receiving trial therapy and underwent surgical resection followed by treatment with bevacizumab. She continued to decline and died one year following her clinical trial enrollment and less than 3 years after initial diagnosis.

### Literature review

**Table 2** summarizes the demographic, diagnostic, treatment, and outcome data of previously published cases of astroblastoma *MN1*-altered and HGNET-MN1 from the 9 studies meeting our inclusion criteria. A total of 30 cases are included in the table (27 previously published plus the three reported in this manuscript). Among these cases, the median age at diagnosis was 9 years with a range of 3.6 to 36 years old. Only six of the 30 patients were diagnosed as adults (18 years of age and older), including 2 of our patients. Only two of the 30 patients were male.

**Table 2:** Demographic, treatment, and outcome data of patients with *MN1*-altered CNS tumors.

Citation	Patient	Age at diagnosis (years)	Sex	Pathological diagnosis (morphological)	Initial treatment	Method used to diagnose *MN1*-altered CNS tumors	Recurrence	Treatment for recurrence	PFS	OS
(6)	1	6	Female	Anaplastic astroblastoma	GTR	DNA and RNA sequencing, RT-PCR, FISH Patient determined to have MN1:BEND2 fusion	Yes	Multiple resections; RT and TMZ for 2nd recurrence; CCNU/TMZ for 3rd recurrence.	7 Months	10 Years
(7)	2	9	Female	Recurrent astroblastoma	SR	DNA Methylation, FISH	Yes	RT	Unknown	+27 Years
(7)	3	10	Female	Anaplastic astroblastoma	SR with RT and TMZ	DNA Methylation, FISH	No	N/A	+2.8 Years	+2.8 Years
(7)	4	31	Female	Malignant glioma, features suggestive of astroblastoma	SR with RT and TMZ	DNA Methylation, FISH	No	N/A	+1 Year	+1 Year
(8)	5	4	Female	Low-grade astroblastoma	GTR	DNA Methylation	No	N/A	+21 Months	+21 Months
(9)	6	4.8	Female	“Four of 10 supratentorial lesions were initially diagnosed morphologically as anaplastic ependymoma. Other diagnoses included peripheral primitive neuroectodermal tumors (one), embryonal tumor not otherwise specified (NOS) (one), atypical glial neoplasia (two), malignant tumor with rhabdoid characteristics (one), and astroblastoma (two). All three spinal tumors were morphologically consistent with ependymoma.” **	GTR with RT (Focal, 59.4 Gy)	Genome-wide methylation arrays/RT-PCR 2 patients determined to have MN1:BEND2 fusion **	No	N/A	+11 Months	+11 Months
(9)	7	5.8	Female	see above “**”	GTR	Genome-wide methylation arrays/RT-PCR see above “**”	No	N/A	+24 Months	+24 Months
(9)	8	8.9	Female	see above “**”	GTR with RT (Focal, 50 Gy) and VAC/VAdriaC	Genome-wide methylation arrays/RT-PCR see above “**”	No	N/A	+9.6 Years	+9.6 Years
(9)	9	5	Female	see above “**”	STR	Genome-wide methylation arrays/RT-PCR see above “**”	Yes	STR with RT (Focal, 45 Gy)	15 Months	+18 Months
(9)	10	4.5	Female	see above “**”	GTR with RT (Focal, 54 Gy)	Genome-wide methylation arrays/RT-PCR see above “**”	Yes	GTR with CP/celecoxib	26.4 Months	35.4 Months
(9)	11	7	Female	see above “**”	GTR with RT (Focal, 59.4 Gy)	Genome-wide methylation arrays/RT-PCR see above “**”	Yes	GTR with RT (CSI, 36 Gy + Boost)	3 Years	6 Years
(9)	12	3.6	Female	see above “**”	STR	Genome-wide methylation arrays/RT-PCR see above “**”	Yes	GTR with RT (Focal, 54 Gy) and VCR/VP/CP	6 Months	+16 Years
(9)	13	6.7	Female	see above “**”	GTR with RT (CSI, 36 Gy + Boost) and SJMB12	Genome-wide methylation arrays/RT-PCR see above “**”	No	N/A	+25 Months	+25 Months
(9)	14	14.6	Male	see above “**”	STR with RT (Focal, 45 Gy)	Genome-wide methylation arrays/RT-PCR see above “**”	Yes	RT (CSI, 36 Gy + Boost) and Oral VP	23 Months	7.3 Years
(9)	15	13	Female	see above “**”	GTR	Genome-wide methylation arrays/RT-PCR see above “**”	No	N/A	+24 Months	+24 Months
(9)	16	36	Male	see above “**”	STR with RT (CyberKnife)	Genome-wide methylation arrays/RT-PCR see above “**”	Yes	STR	5 Years	+11 Years
(9)	17	10	Female	see above “**”	GTR	Genome-wide methylation arrays/RT-PCR see above “**”	No	N/A	+8.3 Years	+8.3 Years
(9)	18	8	Female	see above “**”	GTR with RT (CSI, 23.5 Gy + Boost) and SJMB-96	Genome-wide methylation arrays/RT-PCR see above “**”	Yes	GTR with RT (Focal, 50.4 Gy) and 5D	5.2 Years	+7.5 Years
(9)	19	14	Female	see above “**”	GTR	Genome-wide methylation arrays/RT-PCR see above “**”	No	N/A	+5.3 Years	+5.3 Years
(10)	20	9	Female	Yolk sac carcinoma	STR with chemotherapy	RT-PCR Patient determined to have MN1:BEND2 fusion	Yes	Gamma knife radiosurgery and chemotherapy - GTR following recurrence	8 Months	+12 Months
(11)	21	24	Female	High-grade astroblastoma	GTR with RT (Local, 50 Gy)	FISH Patient determined to have MN1:BEND2 fusion	Yes	GTR	12 Years	+13 Years
(12)	22	36	Female	Low-grade astroblastoma	GTR	Not Reported	Yes	RT for 1^st^ progression STR for 2^nd^ progression Hypo-fractionated stereotactic radiosurgery for 3rd progression, followed by Temozolomide x 6 cycles. GTR for 4th progression followed by bevacizumab and lomustine x 4 cycles.	24 Months	+15 Years
(13)	23	13	Female	Astroblastoma	GTR	Whole Genome Sequencing (WGS) Patient determined to have MN1:GTSE1 and EWSR1:PATZ1 fusions	No	N/A	+36 Months	+36 Months
(14)	24	9	Female	Malignant tumor with papillary and rhabdoid pattern	GTR with RT (Focal, 50 Gy) and VAC/VAdriaC	DNA methylation Patient determined to have MN1:BEND2 fusion	No	N/A	+10 Years	+10 Years
(14)	25	10	Female	Gliofibroma	GTR	RT-PCR Patient determined to have MN1:BEND2 fusion	No	N/A	+2 Years	+2 Years
(14)	26	6	Female	PXA vs ABM; non-typical tumor	GTR	DNA methylation Patient determined to have MN1:BEND2 fusion	No	N/A	+3 Years	+3 Years
(14)	27	4.9	Female	Ependymoma vs ABM vs HGNET-MN1 tumor	GTR with RT (Focal, 59.4 Gy)	RT-PCR Patient determined to have MN1:BEND2 fusion	No	N/A	+2 Years	+2 Years
This Manuscript	28	5	Female	Ependymoma	SR	DNA methylation, RNA sequencing Patient determined to have MN1:BEND2 fusion	Yes	SR for 1st recurrence, RT (59.4 Gy) for 2nd recurrence, GTR for 3rd recurrence.	~24 Months	+21 Years
This Manuscript	29	27	Female	Astroblastoma	SR with RT and TMZ	DNA methylation, RNA sequencing Patient determined to have MN1:BEND2 fusion	No	N/A	+28 Months	+28 Months
This Manuscript	30	33	Female	CNS embryonal tumor with rhabdoid features	SR with RT (30.6 Gy + Boost)	DNA methylation, RNA sequencing Patient determined to have MN1:BEND2 fusion	Yes	GTR, CIS and CCNU, and RT for 1st recurrence. STR for 2nd recurrence with checkpoint inhibitor	~7 Months	2.9 Years

SR, Surgical Resection; GTR, Gross Total Resection; STR, Subtotal Resection; TMZ, Temozolomide; RT, Radiation Therapy; VAC, vincristine-actinomycin D-cyclophosphamide; VAdriaC, vincristine-adriamycin-cyclophosphamide; VCR, vincristine; VP, etoposide; SJMB12, St Jude medulloblastoma regimen; SJMB-96, St Jude medulloblastoma-96 regimen; CP, cyclophosphamide; 5D, five drugs; CIS, cisplatin; CCNU, lomustine; FISH, Fluorescence In Situ Hybridization.

The most common morphological diagnoses prior to a molecular diagnosis of an *MN1*-altered tumor were astroblastoma or ependymoma. During follow up of variable duration, 14 of the 30 patients (46.7%) experienced at least one recurrence of their disease; however, 25 of the 30 (83.3%) were still alive at the time of their last follow up. The shortest progression-free survival (PFS) was 6 months whereas the longest was 12 years. The shortest surviving patient died 35 months (2.9 years) after diagnosis. The longest survival was 27 years, with the patient still alive at the last follow up. We did not identify any other manuscript reporting PROs in patients with this diagnosis.

## Discussion

*MN1*-altered CNS tumors are a newly described and uncommon diagnostic entity. Our NHS, which enrolls adults with primary CNS tumors and routinely incorporates tumor testing with DNA methylation and next generation sequencing panels, identified three participants (all female) with this diagnosis whose comprehensive longitudinal clinical information including PROs, imaging findings and tumor analysis are presented in this manuscript. Two of our patients were diagnosed as young adults, and one experienced a late recurrence as an adult after a prolonged period of stabilization of a tumor diagnosed in her childhood.

Our systematic literature review identified 40 publications of which only 9 provided sufficient information for inclusion. Only four of the 27 cases identified in these 9 publications were diagnosed as adults, indicating the very uncommon presentation in this age group, although this may also reflect a general paucity of advanced molecular testing of primary CNS tumors in adults outside of specialized centers. Few publications discuss the detailed clinical features, management, and outcome of these tumors in adults, and we provide valuable information on the clinical course, diagnosis, and management of this disease.

Our three patients received an integrated diagnosis of HGNET-MN1 before the publication of the latest 2021 WHO classification recommending the term astroblastoma, *MN1*-altered. We found, that despite sharing the same DNA methylation profile and *MN1::BEND2* gene fusion, survival and functional outcomes were quite heterogenous, as reflected by their variable clinical course and our patient’s self-reported symptom burden. Two of our three patients were highly symptomatic, including depression and anxiety and reported an impact on their general health status (one reported this despite the context of prolonged tumor control), highlighting the importance of understanding the patient’s experience in addition to simply analyzing survival data.

Furthermore, the limited existing literature also suggests that outcomes measured as either OS or PFS do not appear to correlate with factors such as age at diagnosis, sex, or initial treatment. Some patients experience a short PFS and multiple recurrences despite treatment with surgery, radiation, and chemotherapy, while others experience years long PFS and OS after only having undergone surgical resection. The determinants of this vast difference in outcomes despite the unifying molecular diagnosis remain unknown and likely include clinical features, tumor genomics and treatment modalities. **Table 2** also highlights a discrepancy between PFS and OS as many patients experience short PFS yet prolonged OS with some experiencing late recurrences (9, 11). Sturm and colleagues reported that of the eight patients diagnosed with HGNET-MN1 in their study, 100% experienced an overall survival (OS) beyond 8 years, but only two had a progression-free survival (PFS) that extended beyond 6 years (2). This discrepancy between PFS and OS was also noted by Lehman and colleagues, who found that patients with MN1-rearranged CNS tumors had a clear and significant survival advantage compared to BRAF^V600E^-mutant tumors, and patients bearing MN1-rearrangements were all alive at the time of publication despite multiple recurrences in some cases (15).

The presence of specific interchromosomal gene fusions may impact survival in patients diagnosed with *MN1*-altered tumors as five of the patients included in our review possessed an *MN1::BEND2* fusion with two of these patients having an OS surpassing 100 months (8.3 years). However, one of our patients with an *MN1::BEND2* fusion (Patient 3) experienced her first recurrence only 7 months after her original diagnosis despite intensive therapy and died in 3 years. We acknowledge that the number of cases with reported gene fusions is very small as a comprehensive molecular profile likely remains unexplored in many patients. Systematic analysis of the presence of specific interchromosomal gene fusions and detailed prospective collection of treatment data may provide further insight into whether specific fusions impact OS and PFS.

Although a specific type of interchromosomal gene fusion may be a contributing factor to these differences in outcome, it is likely not the only one. Of four tumors analyzed using RNA sequencing by Sturm and colleagues, gene fusions between *MN1* and *BEN domain containing 2* (*BEND2*) were observed in three samples (2). An additional gene fusion was observed between *MN1* and *CXXC-type zinc finger protein 5* (*CXXC5*) in another sample (2). High expression levels of BEND2 (a fusion partner) were uniquely observed in HGNET-MN1 tumors distinct from other CNS tumor types (2). Moreover, a set of samples including the tumor harboring the *MN1::CXXC5* fusion formed a distinctly separate cluster *via* t-SNE analysis (t-distributed stochastic neighbor embedding), while all three tumors harboring *MN1::BEND2* fusion were found in a larger homogenous cluster, potentially indicating differences in underlying biology, and therefore outcomes, depending on the *MN1* fusion partner. These findings indicate not only the need for further molecular characterization of these tumors, but also the collection of detailed clinical and treatment data as these may act as other important factors determining outcome.

Currently, only advanced methods such as DNA methylation profiling and targeted next generation sequencing can confirm this diagnosis both in tumors morphologically diagnosed as astroblastomas, and in tumors with histological features that resemble other diagnoses or are insufficient for a specific morphological diagnosis. Hopefully, with the increased recognition of the utility of advanced molecular testing, these tests will be more accessible enabling more accurate diagnosis of a variety of primary CNS cancers. This increase in diagnostic precision will allow better characterization of the natural history of this tumor, ultimately providing important prognostic information and guiding therapy. However, it is essential to collect large series of well annotated and comprehensive clinical, treatment, and outcome data to help decipher prognostic and predictive factors of this and other emergent rare CNS tumors defined by their molecular features.

## Data availability statement

The datasets presented in this study can be found in online repositories. The names of the repository/repositories and accession number(s) can be found in the article/**Supplementary Material**.

## Ethics statement

The three cases discussed in this manuscript were retrieved from enrollees in an IRB-approved Natural History Study at the Neuro-Oncology Branch, National Cancer Institute, National Institutes of Health (NCT02851706; Natural History of and Specimen Banking for People with Tumors of the CNS). The patients/participants provided their written informed consent to participate in this study. Written informed consent was obtained from the individual(s) for the publication of any potentially identifiable images or data included in this article.

## Author contributions

This study was conceptualized and designed by SF and MP-P. A review of the literature was performed by SF and DC. TA is the PI and AA, NB, AnC, AlC, EG, TK, HL, and JL are the core team of the study from which clinical data was collected. LB, NL, KW, MP-P and MG provided direct clinical care to patients included in this manuscript. The data was analyzed and interpreted by SF, MP-P, EV, KA, DP, MQ, RS, TA and MG. SF and MP-P wrote the initial draft of the manuscript. SF, ZA and MP-P created the figures for the manuscript, and MP-P supervised the study. All coauthors reviewed and agreed with the final manuscript. All authors contributed to the article and approved the submitted version.

## Supplementary material

Supplementary Figure 1 Copy number variations obtained *via* DNA methylation analysis in patients 1 (A), 2 (B), and 3 (C). A range of copy number patterns were observed in these tumors. Patient 3 showed both whole-chromosome and partial chromosome gains and losses. Patient 3 also showed alterations in chromosome 22q and X, possibly related to a chromosomal rearrangement leading to MN1::BEND2 fusion.

Supplementary Figure 2 PRISMA Flow Diagram Highlighting Included and Excluded Studies. A total of 83 citations were identified from four databases using the search terms HGNET-MN1, astroblastoma, and MN1-altered. 43 of these citations were removed due to being duplicated. 40 studies were assessed for eligibility. 28 studies were excluded due to there being a lack of molecular and/or clinical data (reason 1), and 3 studies were excluded due to the tumor being an extracranial lesion (reason 2). Of the 40 studies assessed for eligibility, a total of 9 were included in our review.
