# Supplementary material for: Heterogeneous clinicopathological findings and patient-reported outcomes in adults with MN1-altered CNS tumors: A case report and systematic literature review
Source: Front Oncol. 2023 Jan 19;13:1099618. doi: 10.3389/fonc.2023.1099618 (PMC9892899; doi:10.3389/fonc.2023.1099618)
Supplement: Supplementary file 1 [file Table_1.docx]

**Supplementary Table 1:** PROMIS Anxiety and PROMIS depression measurement of anxiety and depression for Patients 1–3.

|  | **Patient 1** | **Patient 2** | **Patient 3** |
| --- | --- | --- | --- |
| Timing | Surveillance | Surveillance | Treatment initiation |
| Depression *t*-score | 61.6 | 38.2 | 64.1 |
| Moderate-severe depressive symptoms | Yes | No | Yes |
| Anxiety *t*-score | 58.3 | 43.1 | 57.1 |
| Moderate-severe anxiety symptoms | No | No | No |
|  |  |  |  |
