# Supplementary material for: Heterogeneous clinicopathological findings and patient-reported outcomes in adults with MN1-altered CNS tumors: A case report and systematic literature review
Source: Front Oncol. 2023 Jan 19;13:1099618. doi: 10.3389/fonc.2023.1099618 (PMC9892899; doi:10.3389/fonc.2023.1099618)
Supplement: Supplementary file 2 [file Table_2.docx]

**Supplementary Table 2:** NeuroQOL Cognition Function measurement of perceived cognition for Patients 1–3.

|  | **Patient 1** | **Patient 2** | **Patient 3** |
| --- | --- | --- | --- |
| Timing | Surveillance | Surveillance | Treatment initiation |
| Cognition function *t*-score | 48.7 | 44.7 | 37.9 |
| Moderate-severe cognition function | No | No | Yes |
|  |  |  |  |
