# Supplementary material for: Heterogeneous clinicopathological findings and patient-reported outcomes in adults with MN1-altered CNS tumors: A case report and systematic literature review
Source: Front Oncol. 2023 Jan 19;13:1099618. doi: 10.3389/fonc.2023.1099618 (PMC9892899; doi:10.3389/fonc.2023.1099618)
Supplement: Supplementary file 3 [file Table_3.docx]

**Supplementary Table 3:** EQ-5D-3L measurement of general health status in Patients 1–3.

|  | **Patient 1** | **Patient 2** | **Patient 3** |
| --- | --- | --- | --- |
| Timing | Surveillance | Surveillance | Treatment initiation |
| Mobility | Some problems in walking about | No problems in walking about | Some problems walking about |
| Self-care | Some problems in washing or dressing | No problems washing or dressing | No problems washing or dressing |
| Usual activities | No problems with usual activities | No problems with usual activities | Some problems with usual activities |
| Pain/discomfort | Moderate pain or discomfort | No pain or discomfort | Moderate pain or discomfort |
| Anxiety/depression | Moderately anxious or depressed | Not anxious or depressed | Moderately anxious or depressed |
| Health state | 22122 | 11111 | 21222 |
| Index score | 0.67 | 1.00 | 0.71 |
