# Supplementary material for: Heterogeneous clinicopathological findings and patient-reported outcomes in adults with MN1-altered CNS tumors: A case report and systematic literature review
Source: Front Oncol. 2023 Jan 19;13:1099618. doi: 10.3389/fonc.2023.1099618 (PMC9892899; doi:10.3389/fonc.2023.1099618)
Supplement: Supplementary file 4 [file Table_4.docx]

*Tumor Testing*

All tumor specimens were reviewed histologically, and molecular analysis was performed at the Laboratory of Pathology, National Cancer Institute, Bethesda, Maryland. Genomic DNA and total RNA were extracted from formalin-fixed paraffin-embedded (FFPE) tissue sections mounted on slides, after macro-dissection to enrich for viable tumor content, if indicated, using the All Prep DNA/RNA FFPE kit (Qiagen). The DNA was bisulfite converted using EZ DNA Methylation kit (Zymo Research) and subsequently processed using Infinium MethylationEPIC kit according to manufacturer’s protocol (Illumina). The beadchip was scanned on iScan reader (Illumina). The signal processing, methylation-based tumor classification, and MGMT promoter methylation analysis were carried out using in house Tumor Classifier software pipeline. The RNA was reverse transcribed into complementary DNA (cDNA) using degenerate hexamer primers. An amplicon-based sequencing library was generated separately from genomic DNA and cDNA by multiplex PCR using a Primary CNS Tumor panel or an Oncomine Comprehensive Assay v3 panel.^9,10^

The Primary CNS Tumor panel is an AmpliSeq custom panel (Thermo Fisher Scientific) of 858 PCR primer pairs (genomic DNA) and 69 primer pairs (cDNA) directed to detect small nucleotide variants in 52 genes, copy number variants (CNVs) in 21 genes, and

fusions between 25 gene pairs that have been associated with CNS cancer pathogenesis (see reportable gene list in supplementary table 4).

Oncomine Comprehensive Assay v3 (Thermo Fisher Scientific) is a panel of 3781 PCR primer pairs (genomic DNA) and 874 primer pairs (cDNA) directed to detect small nucleotide variants (SNVs) in 146 genes, CNVs in 102 genes, and fusions involving 50 genes that have been associated with tumor pathogenesis (see reportable gene list in supplementary table 5). The libraries were pooled and subjected to next generation sequencing using an Ion S5TM XL Sequencing System (Thermo Fisher Scientific). Signal processing, base calling, and alignment to the GRCh37/hg19 human genome assembly were carried out using Torrent SuiteTM software packages (Thermo Fisher Scientific). Variant annotation and interpretation were performed with Ion Reporter Software v.5.10 (Thermo Fisher Scientific). All variants were manually reviewed by visualizing the raw sequencing read alignments using the Integrative Genomics Viewer (Broad Institute, Cambridge, MA). Final interpretation of variants was based on an integration of data from multiple bioinformatics databases (e.g., COSMIC, OMIM, HGMD, ClinVar, gnomAD), protein function prediction tools (e.g., FATHMM, SIFT, PolyPhen-2), and experimental and clinical data reported in the biomedical literature. Variants are reported as significant, likely significant, and variant of uncertain significance based on laboratory developed criteria.^9,10^

Final interpretation of each case was based on integration of methylation-based classification, histo-pathological findings, clinical history, and data reported in biomedical literature.

Supplementary Table 4: Primary CNS Tumor Panel

Small Nucleotide Variants

| ACVR1 | AKT1 | ATRX | BRAF | CCND2 | CCND3 | CDK4 | CDK6 | CDKN2A |
| --- | --- | --- | --- | --- | --- | --- | --- | --- |
| CHEK1 | CHEK2 | CIC | CTNNB1 | DDX3X | EGFR | FGFR1 | FUBP1 | H3F3A |
| HIST1H3B | HIST1H3C | HRAS | IDH1 | IDH2 | IGF1R | KLF4 | KRAS | MDM2 |
| MET | MSH6 | MYC | MYCN | NF1 | NF2 | NOTCH1 | NRAS | NTRK1 |
| NTRK2 | NTRK3 | PARP1 | PDGFRA | PIK3CA | PIK3R1 | PPM1D | PTCH1 | PTEN |
| RB1 | SETD2 | SIRT2 | SMARCA4 | SMARCB1 | SMO | SOX2 | SUFU | TERT |
| TP53 | TRAF7 |  |  |  |  |  |  |  |

Copy Number Variants

| CCND2 | CCND3 | CDK4 | CDK6 | CDKN2A | EGFR | FGFR1 | IGF1R | MET |
| --- | --- | --- | --- | --- | --- | --- | --- | --- |
| MYC | MYCN | NTRK1 | NTRK2 | NTRK3 | PARP1 | PDGFRA | PPM1D | PTEN |
| SOX2 | SUFU | TRAF7 |  |  |  |  |  |  |

Gene Fusions

| BTBD1 : NTRK3 | C11orf95 : RELA | C11orf95 : YAP1 | CLCN6 : BRAF | EGFRvIII |
| --- | --- | --- | --- | --- |
| EGFR : PSPH | EGFR : SEPT14 | ETV6 : NTRK3 | FAM131B : BRAF | FGFR1 : TACC1 |
| FGFR3 : TACC3 | GNAI1 : BRAF | GOPC : ROS1 | KIAA1549 : BRAF | MKRN1 : BRAF |
| MYB : QKI | NAB2 : STAT6 | NACC2 : NTRK2 | QKI : NTRK2 | RELA : RELA |
| RNF130 : BRAF | SRGAP3 : RAF1 | TPM3 : NTRK1 | YAP1 : FAM118B | YAP1 : MAMLD1 |

Supplementary Table 5: Oncomine Comprehensive Assay v3

Small Nucleotide Variants

| AKT1 | AKT2 | AKT3 | ALK | AR | ARAF | ARID1A | ATM | ATR |
| --- | --- | --- | --- | --- | --- | --- | --- | --- |
| ATRX | AXL | BAP1 | BRAF | BRCA1 | BRCA2 | BTK | CBL | CCND1 |
| CCND2 | CCND3 | CCNE1 | CDK2 | CDK4 | CDK6 | CDK12 | CDKN1B | CDKN2A |
| CDKN2B | CHEK1 | CHEK2 | CREBBP | CSF1R | CTNNB1 | DDR2 | EGFR | ERBB2 |
| ERBB3 | ERBB4 | ERCC2 | ESR1 | EZH2 | FANCA | FANCD2 | FANCI | FBXW7 |
| FGF3 | FGF19 | FGFR1 | FGFR2 | FGFR3 | FGFR4 | FLT3 | FOXL2 | GATA2 |
| GNA11 | GNAQ | GNAS | H3F3A | HIST1H3B | HNF1A | HRAS | IDH1 | IDH2 |
| IGF1R | JAK1 | JAK2 | JAK3 | KDR | KIT | KNSTRN | KRAS | MAGOH |
| MAP2K1 | MAP2K2 | MAP2K4 | MAPK1 | MAX | MDM2 | MDM4 | MED12 | MET |
| MLH1 | MRE11A | MSH2 | MSH6 | MTOR | MYC | MYCL | MYCN | MYD88 |
| NBN | NF1 | NF2 | NFE2L2 | NOTCH1 | NOTCH2 | NOTCH3 | NRAS | NTRK1 |
| NTRK2 | NTRK3 | PALB2 | PDGFRA | PDGFRB | PIK3CA | PIK3CB | PIK3R1 | PMS2 |
| POLE | PPARG | PPP2R1A | PTCH1 | PTEN | PTPN11 | RAC1 | RAD50 | RAD51 |
| RAD51B | RAD51C | RAD51D | RAF1 | RB1 | RET | RHEB | RHOA | RICTOR |
| RNF43 | ROS1 | SETD2 | SF3B1 | SLX4 | SMAD4 | SMARCA4 | SMARCB1 | SMO |
| SPOP | SRC | STAT3 | STK11 | TERT | TOP1 | TP53 | TSC1 | TSC2 |
| U2AF1 | XPO1 |  |  |  |  |  |  |  |

Copy Number Variants

| AKT1 | AKT2 | AKT3 | ALK | AR | ARID1A | ATM | ATR | ATRX |
| --- | --- | --- | --- | --- | --- | --- | --- | --- |
| AXL | BAP1 | BRAF | BRCA1 | BRCA2 | CCND1 | CCND2 | CCND3 | CCNE1 |
| CDK2 | CDK4 | CDK6 | CDK12 | CDKN2A | CDKN2B | CHEK1 | CREBBP | CTNNB1 |
| DDR2 | EGFR | ERBB2 | ESR1 | FANCA | FANCD2 | FANCI | FBXW7 | FGF3 |
| FGF19 | FGFR1 | FGFR2 | FGFR3 | FGFR4 | FLT3 | HIST1H3B | HNF1A | IGF1R |
| KIT | KRAS | MDM2 | MDM4 | MET | MLH1 | MRE11A | MSH2 | MSH6 |
| MTOR | MYC | MYCL | MYCN | NBN | NF1 | NF2 | NOTCH1 | NOTCH2 |
| NOTCH3 | NTRK1 | NTRK2 | NTRK3 | PALB2 | PDGFRA | PDGFRB | PIK3CA | PIK3CB |
| PIK3R1 | PMS2 | POLE | PPARG | PTCH1 | PTEN | RAD50 | RAD51 | RAD51B |
| RAD51C | RAD51D | RB1 | RET | RICTOR | RNF43 | ROS1 | SETD2 | SLX4 |
| SMARCA4 | SMARCB1 | SRC | STAT3 | STK11 | TERT | TOP1 | TP53 | TSC1 |
| TSC2 | U2AF1 | XPO1 |  |  |  |  |  |  |

Gene Fusions

| AKT2 | ALK | AR | AXL | BRAF | BRCA1 | BRCA2 | CDKN2A | EGFR |
| --- | --- | --- | --- | --- | --- | --- | --- | --- |
| ERBB2 | ERBB4 | ERG | ESR1 | ETV1 | ETV4 | ETV5 | FGFR1 | FGFR2 |
| FGFR3 | FGR | FLT3 | JAK2 | KRAS | MDM4 | MET | MYB | MYBL1 |
| NF1 | NOTCH1 | NOTCH4 | NRG1 | NTRK1 | NTRK2 | NTRK3 | NUTM1 | PDGFRA |
| PDGFRB | PIK3CA | PPARG | PPKACA | PPKACB | PTEN | RAD51B | RAF1 | RB1 |
