# Supplementary figures and images for: Heterogeneous clinicopathological findings and patient-reported outcomes in adults with MN1-altered CNS tumors: A case report and systematic literature review
Source: Front Oncol. 2023 Jan 19;13:1099618. doi: 10.3389/fonc.2023.1099618 (PMC9892899; doi:10.3389/fonc.2023.1099618)

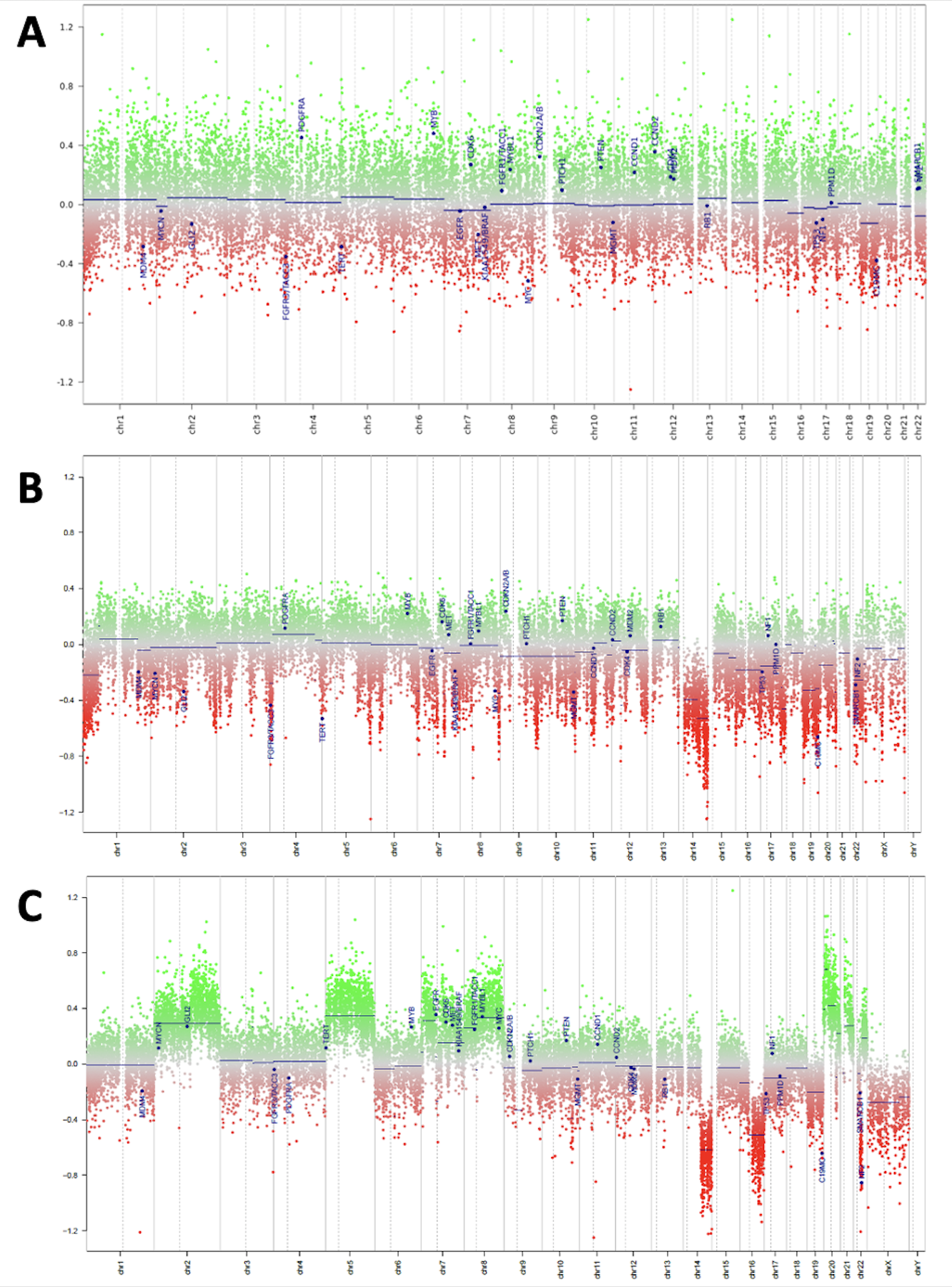

Supplement: Supplementary Figure 1 — Copy number variations obtained via DNA methylation analysis in patients 1 (A), 2 (B), and 3 (C). A range of copy number patterns were observed in these tumors. Patient 3 showed both whole-chromosome and partial chromosome gains and losses. Patient 3 also showed alterations in chromosome 22q and X, possibly related to a chromosomal rearrangement leading to MN1::BEND2 fusion. [file Image_1.tiff]
