# Supplementary material for: Heterogeneous clinicopathological findings and patient-reported outcomes in adults with MN1-altered CNS tumors: A case report and systematic literature review
Source: Front Oncol. 2023 Jan 19;13:1099618. doi: 10.3389/fonc.2023.1099618 (PMC9892899; doi:10.3389/fonc.2023.1099618)
Supplement: Supplementary Figure 2 — PRISMA Flow Diagram Highlighting Included and Excluded Studies. A total of 83 citations were identified from four databases using the search terms HGNET-MN1, astroblastoma, and MN1-altered. 43 of these citations were removed due to being duplicated. 40 studies were assessed for eligibility. 28 studies were excluded due to there being a lack of molecular and/or clinical data (reason 1), and 3 studies were excluded due to the tumor being an extracranial lesion (reason 2). Of the 40 studies assessed for eligibility, a total of 9 were included in our review. [file DataSheet_1.docx]

**Identification of studies via databases**

Records removed *before screening*:

Duplicate records removed

(n = 43)

Records identified from:

PubMed (n = 16)

EMBASE (n=36)
Web of Science (n = 18)
Scopus (n=13)

**Identification**

Records screened

(n = 40)

Records excluded

(n = 0)

Reports sought for retrieval

(n = 40)

Reports not retrieved

(n = 0)

**Screening**

Reports assessed for eligibility

(n = 40)

Reports excluded:

Reason 1 (n = 28)

Reason 2 (n = 3)

Studies included in review

(n = 9)

**Included**

*
